# Causes of Disease

**Published:** 1859-10

**Authors:** 


### CAUSES OF DISEASE.

The complaints of people are in a measure innumerable; every now and then a peculiarity of ailment is presented which is not recorded in any book extant; just as new questions of law are constantly arising. But while the effects of disease are so numerous, the causes of them may be reduced down so low as to be all told in the number five:

*First*—Poisons.

*Second*—Improper eating.

*Third*—Variations of atmosphere.

*Fourth*—Occupations.

*Fifth*—Hereditary tendencies; which last indeed is a modification of the first.

Of the four, by far the most frequent causes of disease are found in the food we eat, and in the air we breathe, the rectification of both of which is within our own power; requiring only a moderate amount of intelligence, but a large share of moral power, that is, a resolute self-denial. It thus follows, that death, short of old age, is chargeable to man himself; that in an important sense, the great mass of those who die short of threescore years and ten, are the authors of their own destruction. And each should inquire "to what extent am I chargeable with my own ailments?"

